# Effects of pre-adult photoperiod experience on reproductive parameters of *Chrysoperla nipponensis* (Tjeder): potential implications for mass-rearing of natural enemies

**DOI:** 10.3389/finsc.2025.1680910

**Published:** 2025-09-25

**Authors:** Xue Kong, Minghui Xu, Haolin Li, Shaofeng Zhong, Dandan Li, Yongyu Xu, Zhenzhen Chen

**Affiliations:** ^1^ State Key Laboratory of Wheat Improvement, College of Plant Protection, Shandong Agricultural University, Tai’an, China; ^2^ Jiaodong Innovation Center, Shandong Institute of Sericulture, Shandong Academy of Agricultural Sciences, Yantai, China

**Keywords:** photoperiod, reproduction, diapause, green lacewing, fecundity

## Abstract

Photoperiod is a critical environmental factor for insect development and physiology, yet little is known about the effects of photoperiodic signals received during photoperiod-sensitive stages on reproductive parameters. The green lacewing, *Chrysoperla nipponensis*, is a promising candidate for mass rearing in biological control. Photoperiod is the primary environmental factor influencing *C. nipponensis* reproductive diapause. This study investigates how photoperiodic cues during photoperiod-sensitive stages affect key reproductive parameters such as fecundity, lifespan, oviposition duration, oviposition rate, diapause rate, pre-oviposition period, and lipid content of *C. nipponensis*. The results showed that short-day conditions (Light:Dark = 9h:15h; L9:D15) during pre-adult stages increase total lipid and triglyceride levels in both third larvae and newly emerged females, thereby enhancing fecundity of female, without reducing lifespan or oviposition. Furthermore, long-day conditions (Light:Dark = 15h:9h; L15:D9) during the pre-adult stage inhibited diapause, while increasing fecundity and extending oviposition duration. Our findings demonstrate that photoperiodic signals during the pre-adult stages significantly affect the reproductive parameters of *C. nipponensis*, which advances the understanding of photoperiod-dependent reproductive diapause and offers novel insights for optimizing strategies in mass-rearing of natural enemies.

## 1 Introduction

Green lacewings are widely distributed predatory natural enemies (1–3). Due to their ability to enter diapause, broad prey range, high predation rate and ease of mass-rearing, green lacewings are commercialized and widely used for biological control in various agricultural ecosystems (4–6). To enhance the effectiveness of green lacewings as biological control agents, studies focused on the effects of artificial diets on their reproductive success (7, 8), and others investigated optimal conditions for the cold storage (9, 10). However, the balance between the storage and reproduction in natural enemy insects is crucial for maintaining their supply capacity (4).

The occurrence of diapause provides a natural strategy to facilitate the long-term storage (11). Diapause is a seasonal adaptation in insects, evolved to withstand adverse environmental conditions (12). During diapause, physiological activities such as growth, development, and reproduction are significantly suppressed (13–15). In addition, insects store more nutrients, such as lipids, to prepare for diapause (16, 17). The low metabolism and sufficient nutrient during diapause contributes to extending the lifespan of insects (18, 19).

Photoperiod is a key environmental factor influencing insect diapause (12, 20). Additionally, photoperiod influences the reproductive parameters of insects (21–23). In *Holotrichia oblita*, as the duration of the dark period increases, the pre-oviposition period shortens, while the oviposition period is extended (24). Otherwise, some insects exhibit the highest fecundity under relatively long photoperiods (21, 25, 26). Therefore, for diapause-capable insects, photoperiod not only directly influences the diapause process but may also affect reproductive parameters.

The green lacewing, *Chrysoperla nipponensis*, enters diapause as adults, with photoperiod being the primary environmental factor for the induction and maintenance of diapause (27). The third instar larvae, pre-pupae and adult stages of *C. nipponensis* are all sensitive for photoperiod (28). Reproductive parameters, including fecundity, pre-oviposition period, oviposition period and lifespan, are crucial for developing efficient mass-rearing systems (29, 30). A thorough analysis of *C. nipponensis* reproductive parameters under varying photoperiods at different developmental stages is essential. Here, we systematically analyzed the effects of long-day and short-day conditions during the larval, pupal and adult stages on *C. nipponensis.* The results will provide a theoretical basis of enhancing the reproductive potential and developing large-scale artificial breeding for natural enemy insects.

## 2 Materials and methods

### 2.1 Insect collection and rearing

The *C. nipponensis* used in this study was sourced from a stable colony maintained at the Insect Physiology and Ecology Laboratory of Shandong Agricultural University. Eggs were collected by clipping the egg stalks and transferring them to sterile disposable plastic Petri dishes (90 mm × 15 mm). A sufficient quantity of sterilized eggs from *Corcyra cephalonica* was placed in the dishes as a food source for newly hatched larvae to prevent cannibalism and ensure synchronous development. Then, larvae were individually transferred into cylindrical glass tubes (1 cm in diameter, 7 cm in height). Within these tubes, larvae were fed *Megoura japonica* reared on broad bean (*Vicia faba*) plants until pupation. Within 12 hours of adult emergence, male and female adults were paired and housed in canning jars (8 cm in diameter and 10 cm in height). They were fed an artificial diet of yeast powder and powdered sucrose in a 10:8 mass ratio (sucrose ground and sieved through a 60-mesh filter), supplemented with a 10% honey solution. Long-day (LD) conditions were set a 15:9 h light/dark photoperiod, while short-day (SD) conditions were set at a 9:15 h light/dark photoperiod. All rearing conditions were maintained at 25 ± 1 °C and 70 ± 5% relative humidity.

### 2.2 Quantification of reproductive traits and female lifespan

**Figure 1:**
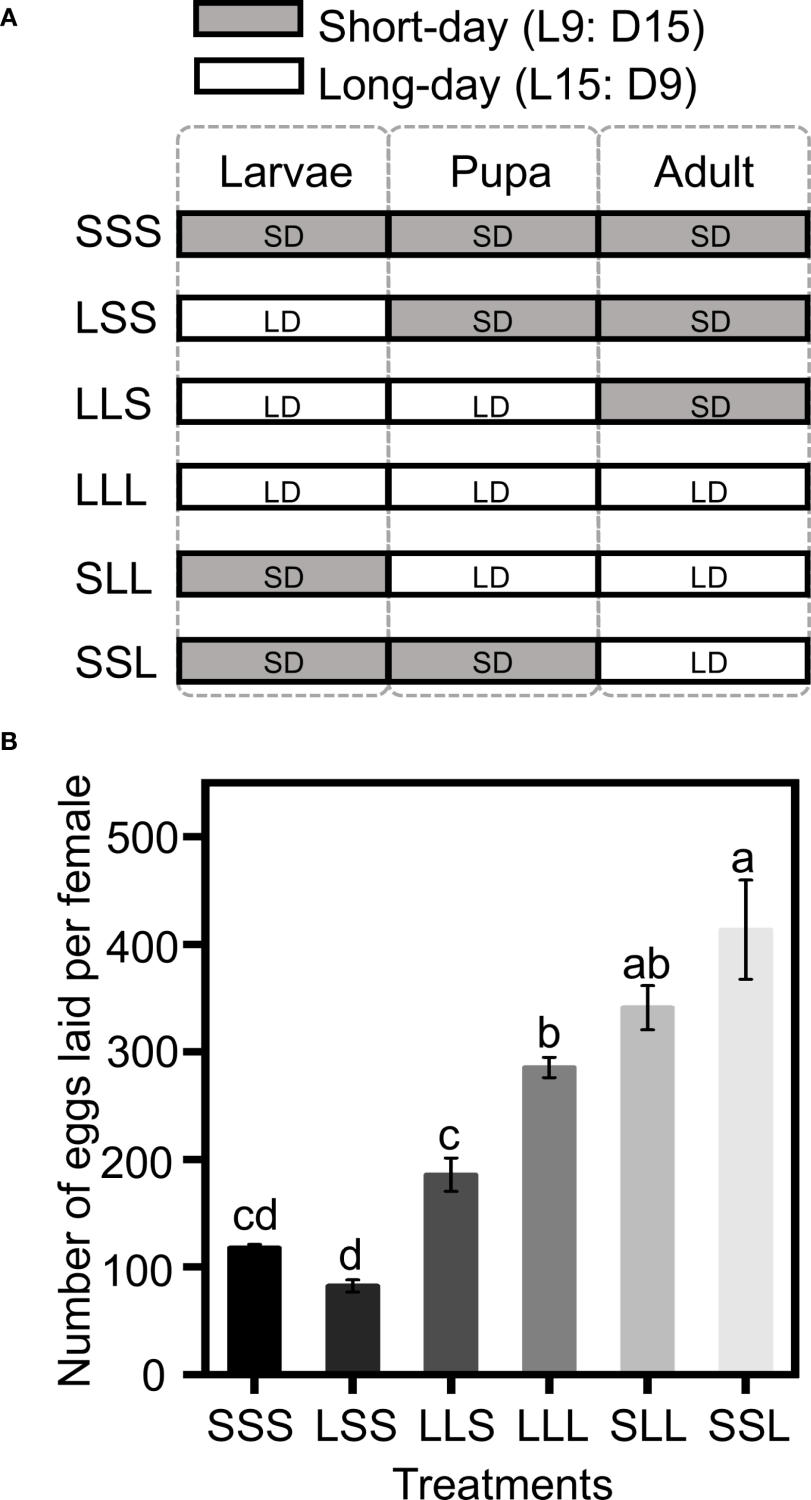
Fecundity with varying photoperiod treatments. **(A)** Experimental group design diagram. The photoperiod treatments (SSS, SSL, SLL, LLL, LLS, and LSS) were applied to six groups, with each group receiving a specific combination of long-day and short-day conditions for the larval, pupal, and adult stage. The X-axis denotes the developmental stages. The Y-axis represents the photoperiod treatments. SD (short-day condition, L9:D15), LD (long-day condition, L15:D9). **(B)** Number of eggs laid per female. Data in the figure are presented as Mean ± SEM. Different lowercase letters indicate significant differences among multiple groups according to one-way ANOVA followed by Tukey’s *post hoc* test for multiple comparisons, *P* < 0.05. n = 30 from 3 independent biological replicates.

Newly hatched larvae were divided into six groups (LLL, LLS, LSS, SSS, SSL, and SLL), with larval, pupal, and adult stages exposed to either long-day or short-day conditions in incubators. The treatments are as follows (**Figure 1**): the LLL group, where the larval, pupal, and adult stages were exposed to long-day conditions; the LLS group, where the larval and pupal stages were exposed to long-day conditions, and the adult stage to short-day conditions; the LSS group, where the larval stage was exposed to a long-day conditions, and the pupal and adult stages to short-day conditions; the SSS group, where the larval, pupal, and the adult stages were exposed to short-day conditions; the SSL group, where the larval and pupal stages were exposed to short-day conditions and the adult stage to long-day conditions; the SLL group, where the larval stage was exposed to short-day conditions, and the pupal and the adult stages to long-day conditions.

To analyze reproductive traits and female lifespan of *C. nipponensis* under varying photoperiod treatments, the reproductive behavior and developmental progress of female adults were observed and recorded daily until all individuals died. The following parameters were calculated for each treatment: the number of days from adult emergence to the onset of oviposition (pre-oviposition period); the number of days from the beginning to the end of oviposition (oviposition duration); the number of eggs laid by a single female adult (fecundity); and the number of days from female adult emergence to death (lifespan). Each treatment was replicated 3 times, and each replicate included 10 females.

### 2.3 Measurement of total lipid and triglyceride content

To investigate the mechanisms underlying photoperiod effects on the fecundity of *C. nipponensis*, total lipid and triglyceride content of third-instar larvae and newly emerged females were measured. The third-instar larvae were reared under constant long-day or short-day conditions from first instar, while newly emerged females were exposed to either long-day or short-day conditions from first instar to eclosion. Each treatment was replicated 4 times, and each replicate included 5 individuals. The total lipid content was determined using the chloroform-methanol extraction method as described by Chen et al. (27). Samples were collected and dried in electrothermal drying oven (GZX-9240MBE, BoXun, China) at 60 °C for 72 h. After drying, dry weight (DW) was measured using the electronic microbalance (A200S, Sartorius, Germany). Meanwhile, *C. nipponensis* was transferred into a new centrifuge tube, weighed (W1), and then homogenized in 500 μL of chloroform/methanol (2:1, v:v). Samples were centrifuged at 2600 g for 10 min, and the pellets were retained. The process was repeated, and the samples were dried at 60 °C for another 72 h. The dried samples weights (W2) were measured, and the total lipid content was calculated as (W1 − W2)/DW.

Triglyceride content was measured using the liquid triglycerides GPO-PAP method. Weigh the samples and add anhydrous ethanol at a ratio of 1:9 (weight (g) to volume (mL)). Homogenize the samples thoroughly on ice using a motorized homogenizer. Centrifuge at 2,500 rpm for 10 min. The supernatant was used to determine triglyceride content using Triglyceride assay kit (code A110-2-1, Nanjing Jiancheng Institute of Bioengineering, China). Measure the absorbance of each tube at a wavelength of 510 nm using a microplate reader (SYNERGYMx, BioTeK, USA). Triglyceride content (μmol/g) = (Sample OD − Blank OD)/(Calibration OD − Blank OD) × Concentration of Sample.

### 2.4 Adult reproductive status

To define female adults reproductive status under varying photoperiod treatments, oviposition rate, non-oviposition rate, diapause rate and non-diapause rate were assessed 15 days post-eclosion according to the criteria established by Xu et al. (28). Adults that laid eggs and those which did not lay eggs but their ovaries were at developmental stages III or IV were classified as non-diapause. Adults with less developed ovaries were classified as diapause. The photoperiod treatments are divided into six groups (LLL, LLS, LSS, SSS, SSL, and SLL), as described earlier. Each treatment was replicated 3 times, and each replicate included 10 females.


$$Diapause\;Rate\;\left(\%\right)=\frac{\text{Number of diapause individuals}}{\text{Total number of individuals}}\times 100\%$$

$$No\mathrm{n‐}diapause\;Rate\;\left(\%\right)=\frac{\text{Number of nondiapause individuals}}{\text{Total number of individuals}}\times 100\%$$

$$Oviposition\;Rate\;\left(\%\right)=\frac{\text{Number of egglaying individuals}}{\text{Total number of individuals}}\times 100\%$$

$$No\mathrm{n‐}oviposition\;Rate\;\left(\%\right)=\frac{\text{Number of nonegglaying individuals}}{\text{Total number of individuals}}\times 100\%$$

### 2.5 Statistical analysis

Statistical analysis was performed by GraphPad Prism 8.0 (GraphPad Software, USA). All data are expressed as mean ± standard error of mean (Mean ± SEM). Differences between two groups were assessed using Student’s t-test (two-tailed, * *P* < 0.05, ** *P* < 0.01, *** *P* < 0.001, and **** *P* < 0.0001). Fecundity, female lifespan, oviposition duration, and pre-oviposition period of *C. nipponensis* were compared across varying photoperiod treatments using a one-way ANOVA, followed by Tukey’s *post hoc* test for multiple comparisons (*P* < 0.05).

## 3 Results

### 3.1 Effect of photoperiod treatment on female fecundity

Photoperiod treatments at different developmental stages significantly affected the fecundity of *C. nipponensis.* Groups in which adults were exposed to long-day conditions (LLL, SLL, SSL) had higher fecundity than groups that exposed to short-day conditions (SSS, LSS, LLS) (*F* = 41.280; *df* = 5,174; *P <*0.001) (**Figure 1**). Fecundity was 285.30 ± 20.19 eggs for LLL, 340.93 ± 23.50 eggs for SLL and 413.53 ± 21.00 eggs for SSL. Notably, SSL exhibited the highest fecundity among LLL, SLL, and SSL, with fecundity increasing as the duration of short-day exposure prolonged during pre-adult stages. Among SSS, LSS and LLS, LLS exhibited the highest fecundity (185.70 ± 28.31 eggs), followed by SSS (117.67 ± 12.83 eggs) and LSS (82.30 ± 10.34 eggs).

### 3.2 Effect of photoperiod treatment on female lifespan

**Figure 2:**
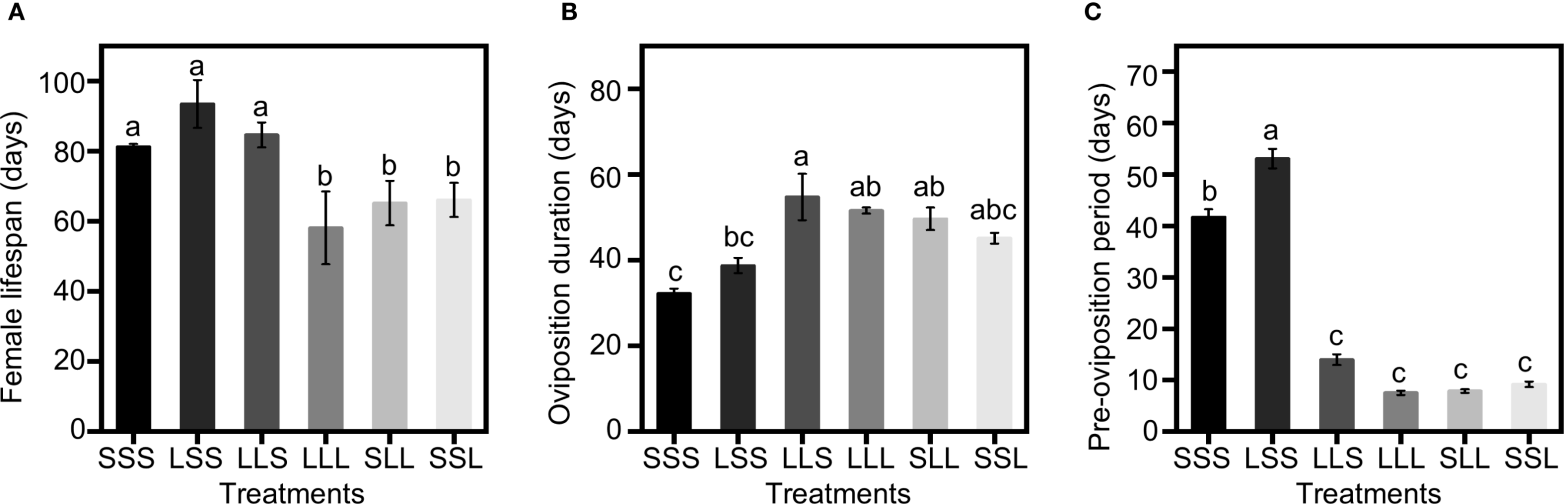
Lifespan, oviposition duration and pre-oviposition period with varying photoperiod treatments. **(A)** Female lifespan. **(B)** Oviposition duration. **(C)** Pre-oviposition period. Data in the figure are presented as Mean ± SEM. Different lowercase letters indicate significant differences among multiple groups according to one-way ANOVA followed by Tukey’s *post hoc* test for multiple comparisons, *P* < 0.05. n = 30 from 3 independent biological replicates.

Photoperiod treatments at different developmental stages significantly influenced the female lifespan of *C. nipponensis*, with females exposed to short-day conditions (SSS, LSS, LLS) are all longer than those exposed to long-day conditions (LLL, SLL, SSL) (*F* = 13.602; *df* = 5,174; *P <*0.001) (**Figure 2A**). The female lifespan of SSS, LSS and LLS was 81.23 ± 2.14 days, 93.47 ± 3.83 days, and 84.63 ± 3.78 days, respectively. The female lifespan of LLL, SLL and SSL were 58.13 ± 3.99 days, 65.20 ± 4.49 days, and 66.07 ± 3.56 days, respectively. In the LLL, SLL, and SSL, female lifespan increased with the duration of exposure to short-day conditions during the pre-adult stages, although no significant differences were observed among the three long-day adult groups.

### 3.3 Effect of photoperiod treatment on oviposition duration

The oviposition duration was significantly influenced by photoperiod treatments at different developmental stages (*F* = 4.849; *df* = 5,174; *P <*0.001) (**Figure 2B**). The LLS had the longest oviposition duration among all groups, at 54.77 ± 4.84 days. The oviposition duration of SSS was 32.20 ± 1.89 days and LSS was 38.77 ± 3.11 days, showing a decreased trend with increased exposure to short-day conditions. For the LLL, SLL and SSL, the oviposition durations were 51.60 ± 4.58 days, 49.67 ± 4.25 days, and 45.23 ± 3.75 days, respectively, with no significant differences among these groups.

### 3.4 Effect of photoperiod treatment on pre-oviposition period

Photoperiod had a significant impact on the pre-oviposition period of *C. nipponensis* (*F* = 136.727; *df* = 5,174; *P <*0.001) (**Figure 2C**). Among SSS, LSS and LLS, LLS had a significantly shorter pre-oviposition period than SSS and LSS. Among LLL, SLL and SSL, SSL had the longest pre-oviposition period (9.07 ± 0.21 days), compared to LLL (7.57 ± 0.28 days) and SLL (7.90 ± 0.29 days). Notably, the pre-oviposition period of LLS did not differ significantly from groups that adult stages exposed to long-day conditions (LLL, SLL, SSL).

### 3.5 Effect of photoperiod treatment on female reproductive status

To further investigate the impact of photoperiod on reproductive status, we assessed the ovarian development and egg-laying behavior of females after varying photoperiod treatments (**Table 1**). Females exposed to long-day conditions (LLL, SLL, SSL) exhibited 0% diapause rate and 100% oviposition rate. In contrast, females under short -day conditions (SSS, LSS) during the adult stage entered diapause with 100% diapause rate and 0% oviposition rate. Additionally, in LLS, 13.33 ± 0.33% of adults entered diapause, while 86.67 ± 0.33% were non-diapausing. Regarding reproductive behavior, 80.00 ± 0.06% of adults laid eggs, whereas 20.00% did not oviposit.

**Table 1:** Adult reproductive status of *C. nipponensis* at different developmental stages under long-day or short-day conditions.

Treatments	Photoperiod condition			Diapause rate (%)	Non-diapause rate (%)	Oviposition rate (%)	Non-oviposition rate (%)
	Larvae	Pupa	Adult				
SSS	SD	SD	SD	100 ± 0	0 ± 0	0 ± 0	100 ± 0
LSS	LD	SD	SD	100 ± 0	0 ± 0	0 ± 0	100 ± 0
LLS	LD	LD	SD	13.33 ± 0.33	86.67 ± 0.33	80.00 ± 0.06	20.00 ± 0.06
LLL	LD	LD	LD	0 ± 0	100 ± 0	100 ± 0	0 ± 0
SLL	SD	LD	LD	0 ± 0	100 ± 0	100 ± 0	0 ± 0
SSL	SD	SD	LD	0 ± 0	100 ± 0	100 ± 0	0 ± 0

The photoperiod treatments (SSS, SSL, SLL, LLL, LLS, and LSS) were applied to six groups, with each group receiving a specific combination of long-day and short-day conditions for the larval, pupal, and adult stage. SD (short-day condition, L9:D15), LD (long-day condition, L15:D9). Data in the table are presented as Mean ± SEM. Each treatment was replicated 3 times, and each replicate included 10 females.

### 3.6 Effect of photoperiod treatment on lipid content

**Figure 3:**
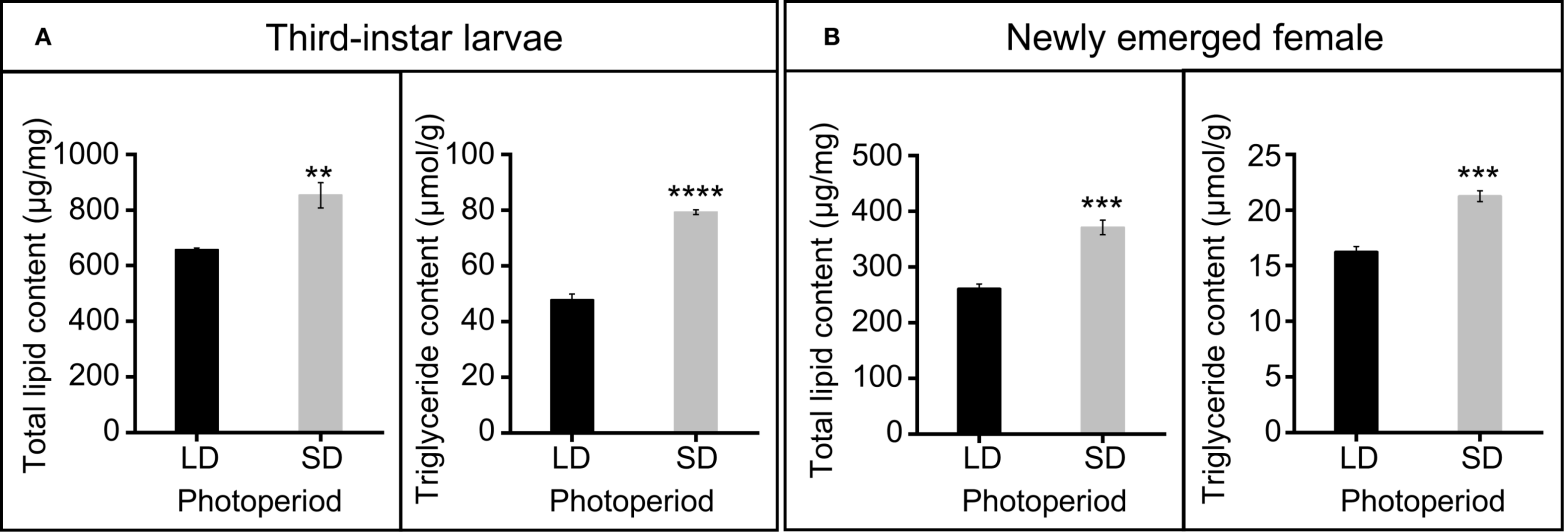
Total lipid and triglyceride content under long-day and short-day conditions. **(A)** Total lipid and triglyceride content of third larvae. **(B)** Total lipid and triglyceride content of newly emerged female. Data in the figure are presented as Mean ± SEM. Differences between two groups were analyzed using Student’s t-test (In A, **P = 0.005, ****P < 0.0001, respectively; In B, ***P = 0.0004, ***P = 0.0003, respectively). SD (short-day condition, L9:D15), LD (long-day condition, L15:D9). n = 4 independent biological replicates.

Photoperiod significantly affected total lipid and triglyceride content in both third-instar larvae and newly emerged females (**Figure 3**). In third-instar larvae, short-day conditions significantly increased total lipid content (*t* = 4.28, *df* = 6, *P* = 0.005), from 656.82 ± 6.93 μg/mg under long-day conditions to 852.70 ± 45.23 μg/mg under short-day conditions (**Figure 3A**). Similarly, triglyceride content also significantly increased from 47.66 ± 2.16 μmol/mg under long-day conditions to 79.23 ± 0.88 μmol/mg under short-day conditions in third-instar larvae (*t* = 13.528, *df* = 6, *P* < 0.0001) (**Figure 3A**). In newly emerged females, total lipid content significantly increased from 261.00 ± 8.23 μg/mg under long-day conditions to 370.67 ± 13.10 μg/mg under short-day conditions (*t* = 7.090, *df* = 6, *P* = 0.0004) (**Figure 3B**). Similarly, triglyceride content significantly increased from 16.24 ± 0.49 μmol/mg under long-day conditions to 21.24 ± 0.48 μmol/mg under short-day conditions (*t* = 7.294, *df* = 6, *P* = 0.0003) (**Figure 3B**).

## 4 Discussion

Insects typically show higher fecundity under long-day conditions than short-day conditions (31–33). For instance, extending the photoperiod beyond 12:12 (light:dark) improves progeny yield in *Telenomus remus* (34). Our study confirms that females exposed to long-day conditions during the adult stage exhibited higher fecundity than those under short-day conditions, regardless of the photoperiod during the pre-adult stages (**Figure 1**). High fecundity is typically at the expense of the lifespan (35). In *C. nipponensis*, we observed shorter lifespan in adult exposed to long-day conditions (LLL, SLL, SSL) than those exposed to short-day conditions (SSS, LSS, LLS). However, among LLL, SLL and SSL, short-day conditions during pre-adult stages significantly increased fecundity (**Figure 1**), without shorten the lifespan and oviposition duration (**Figure 2**). These findings could be utilized to improve fecundity of enemy insects.

Lipid accumulation is a physiological response in insects to diapause-inducing signals (27, 36). Lipid reserves primarily accumulate as triglycerides (37), which are crucial for oogenesis, embryogenesis, and egg maturation (38). The fecundity of insects is associated with lipid content (39–41). Larval and pupal stages of *C. nipponensis* response short photoperiod then increase the lipid content (**Figure 3**), which may be a critical factor contributing to enhanced fecundity. Short-day conditions also significantly affect the expression of genes involved in lipid synthesis and metabolism in *C. nipponensis* larvae (42), providing molecular support for our findings.

As the depth of diapause induction increases, energy is stored and gradually consumed to sustain diapause until termination (43, 44). Adults exposed to short-day conditions have a longer lifespan than those under long-day conditions (**Figure 2**), which benefits the shelf-life of natural enemies, but their fecundity is reduced (**Figure 1**). Diapause development depletes nutrients, thereby reducing the energy available for reproduction (45). Notably, the LLS, exposed to long-day conditions during the pre-adult stage, had a longer oviposition period (**Figure 2**) and higher fecundity compared to SSS (**Figure 1**). Additionally, the LSS, which under long-day conditions during the larval stage, exhibited a longer lifespan than the SSS group (**Figure 2**). However, the optimal photoperiod transition to enhance fecundity and extend the shelf-life of natural enemies remains to be determined.

Diapause is a clear advantage in improve shelf-life and long-distance shipment in biological control (9, 46). The accumulation of diapause stimuli during sensitive stages maximizes the diapause response in *Anastatus japonicus* and *Tetrastichus septentrionalis* (47, 48). In *C. nipponensis* the photoperiod during the adult stage is the most critical factor determining reproductive diapause (28). Although the third-larval and pupal stages are also photoperiod-sensitive, short-day conditions during these stages alone do not induce diapause but extend the pre-oviposition period (**Figure 2**). Moreover, short-day conditions during the pre-adult stage promote diapause whereas long-day conditions reduce diapause incidence (**Table 1**). Similar phenomena have been observed in *Laodelphax striatellus* and *Chrysopa downesi* (49, 50). Therefore, photoperiod signals during pre-adult stages accumulate into the adult stage, influencing reproduction and diapause in *C. nipponensis*. Our findings demonstrated that complete diapause does not necessarily require short photoperiods throughout the entire developmental period. Diapause induction that begins in pupal stage is sufficient to induce 100% diapause of adults in *C. nipponensis*.

In summary, photoperiodic signals during pre-adult stages accumulate and influence reproductive and diapause biology of *C. nipponensis*. Most notably, short-day conditions during the pre-adult stage promote lipid accumulation and enhance fecundity of females. Specifically, the accumulation of short-day conditions stimuli during pupal stage is essential to maximize the diapause response. As is common, the occurrence of diapause facilitates the storage of natural enemy insect products, while balancing reproduction and storage is crucial for commercial applications. Our findings provide insights for advancing fundamental research on reproduction and supporting the large-scale production and application of lacewings.

## Data Availability

The original contributions presented in the study are included in the article/supplementary material. Further inquiries can be directed to the corresponding author.
